# Neural correlates of fanhood: the role of fan identity and team brand strength

**DOI:** 10.3389/fnhum.2023.1235139

**Published:** 2024-01-08

**Authors:** Ricardo Cayolla, Rui Biscaia, Roy F. Baumeister, Hang-Yee Chan, Isabel C. Duarte, Miguel Castelo-Branco

**Affiliations:** ^1^Department of Economics and Management, Consumer Neuroscience Lab, REMIT, Portucalense University, Porto, Portugal; ^2^Department for Health, Faculty of Humanities and Social Sciences, University of Bath, Bath, United Kingdom; ^3^School of Psychology, The University of Queensland, Saint Lucia, QLD, Australia; ^4^Kings College London, London, United Kingdom; ^5^Institute of Nuclear Sciences Applied to Health, Universidade de Coimbra, Coimbra, Portugal

**Keywords:** consumer neuroscience, sport fans, fan identity, brand love, team brands

## Abstract

**Introduction:**

We analyzed the importance of fan identity and brand strength on fans’ neural reactions to different team-related stimuli.

**Methods:**

A total of 53 fMRI scans with fans of two professional sport teams were conducted. Following up on a previous study we focused on the differences between fandom levels as well as the contrast between two team “brand” strength. Neural responses were compared among individuals based on their levels of fan identity. In sum, group comparisons between relatively high and lower identity and between weak and strong teams were made based on the notion that the latter reflects team brand strength (strong brand and weak brand).

**Results:**

Findings indicate that brain activity in emotion regulation, memory, and cognitive control circuits is influenced by the relative level of fan identity.

**Discussion:**

Higher-level identified fans showed increased reactivity to positive stimuli and the under-recruitment of their cognitive appraisal circuits, suggesting more vulnerability to marketers’ messages. The strength of the team brand activates different neural mechanisms. Interestingly, the posterior cingulate showed larger recruitment both for weaker brands and lower fan identification, suggesting that visual memory processes are more active in these cases. Neurally processed content depends on the relative brand’s strength, highlighting the importance of brand-focused communications.

## Highlights

– Findings indicate that brain activity in emotion regulation, memory, and cognitive control circuits is influenced by the degree of fan identification—the stronger identification with the team, the less critical the brand’s strength.
– Higher-level identification fans showed increased reactivity to positive stimuli and the under-recruitment of their cognitive control circuits, suggesting more vulnerability to marketers’ messages.
– The strength of the team brand activates different neural mechanisms.
– Neurally processed content depends on the brand’s strength, highlighting the importance of brand-focused communications.

## Introduction

In the sport industry, fans are undeniably key stakeholders (García and Welford, 2015) because they invest time, money and effort in supporting the teams and are the final consumers of the services offered by teams either directly (i.e., live events) or indirectly (i.e., TV viewers and target of sponsors), while also influencing organizational decision-making (Senaux, 2008).

For example, the strong opposition of football fans to the creation of the European Super League (ESL) led to its collapse a few days after the announcement, with the ESL proponent teams changing their plans to please their strongly identified fans instead of commercial reasons (The Guardian, 2021). Fan identity (i.e., the importance of the role of being a fan to the individual; Biscaia et al., 2018) is a key aspect in the relationship team-fan acting as a predictor of future behaviors (Lock and Heere, 2017). Notwithstanding, sport fans show variable characteristics (Ross, 2007) and it is important to understand their nuanced reactions to teams. Fans are devoted individuals with an enduring interest in sport teams or players (Sun et al., 2021) and are often differentiated in terms of the intensity of their identification levels (Davis et al., 2019; James et al., 2019). Also, the strength of team brands often depends on a myriad of both product and non-product related attributes (Gladden and Funk, 2002), and this often affects how fans and teams connect with each other (Doyle et al., 2013). In this vein, it is vital to explore how different fans react neuronally to team brands with different levels of market prominence and on-field success.

Brands are cultural symbols that often provide special meanings for certain consumer groups (Schaefer, 2009; Hedlund et al., 2020; Veloutsou et al., 2020). People process brand information through explicit memory (Keller, 1993), but also implicit memory (Deppe et al., 2005), with both being mediated through the feelings and emotions triggered (Bechara and Damasio, 2005). Previous neural studies with sport consumers have been carried out based on a dichotomy of fans vs. non-fans (Bilgiç et al., 2020), and the results demonstrate a clear difference in neural activation patterns, specifically related to rewards and motivational states. However, the importance of different levels of fan identity on the reaction to team brands is yet to be explored. Also, the consumer behavior literature has focused on neutral, weak, and strong brands, comparing them with respect to familiarity and specific brand associations among consumers in general (e.g., Dahlén and Lange, 2005; Esch et al., 2012). As team brands often enjoy global popularity and fans share strong emotional bonds with them regardless of the on-field success and existent rivalries (Bauer et al., 2005), the current study sought to extend previous work (e.g., Duarte et al., 2017; Bilgiç et al., 2020) by examining how the contrast level of fan identity and the strength of the team brand affect fan neural responses to videos of football matches with goal moments.

In measuring responses to external stimuli, quantitative approaches (e.g., self-report questionnaires) and qualitative approaches (e.g., interviews and focus-groups) have shed valuable light (Ohme et al., 2011) to understand the link consumer-brand. However, these methods are often susceptible to bias, either by the interviewee or the interviewer (Harris et al., 2018). Memory failures, the presence of the interviewers themselves, hiding possible feelings of shame, or lack of precision about feelings and preferences are some of the potential weaknesses (Harrell, 2019). Agarwal and Dutta (2015) propose that neuroscience research can provide a complementary perspective to traditional marketing methods. The most frequently used neuroimaging technique is the functional magnetic resonance imaging (fMRI; Karmarkar et al., 2015b) to understand the brain processing mechanisms of the consumers (Krishnan, 1996; Eser et al., 2011; Duarte et al., 2018).

Although brand strength has been explored in previous research (e.g., Mühlbacher et al., 2016; Li et al., 2019), there is an absence of knowledge regarding neural responses based on fan identity levels and the strength of the team brand in the market. This is important to examine because of the role often attributed to brand strength (Mühlbacher et al., 2016) and fan identity (Biscaia et al., 2018) to promote consumer-brand relationship over time. To fill these gaps, the current study examined the neural correlates of sport fans in relation to two contrast levels of identity (high and very high), as well as how these neural correlates are influenced by the team brand strength (contrast between strong brand and weak brand). We conducted fMRIs in fans of two professional football teams, differing in their level or competitive strength and fan identity. Given the importance of consumer identification with brands and the lack of research on the neural correlates of individuals with high but distinguishable identification levels with a brand, this study aims to contribute the neural and behavioral understanding about the identification and respective strength of the brand.

## Literature review

### Consumer neuroscience and sport fans

Consumer neuroscience techniques (i.e., examination of consumer behavior with neuroscience methods; Goto et al., 2017) help to understand neurobehavioral consumer profiles (Stanton et al., 2017). Neural responses of consumers have been the subject of a growing body of research focusing on consumer-brand relationships (Couwenberg et al., 2020). To this respect, the reward circuitry has been often subject to research to understand cultural objects (Erk et al., 2002), favorite brands (Schaefer and Rotte, 2007a), package design (Reimann et al., 2010), price context (Schmidt et al., 2017), and videos (Chan et al., 2018).

A positive reward provides action reinforcement (Schultz, 2015). The ventral tegmental area (VTA), nucleus accumbens, anterior cingulate, and hypothalamus form a set of neural structures that are implicated in the reward system (Berridge and Kringelbach, 2015). Sport is an emotionally charged environment full of events with potential to trigger multiple fan reactions (Biscaia et al., 2012; Koenig-Lewis et al., 2017). Sport fans feel team victories and defeats as if these were their own, so it is with this in-group bias needed to be understood in terms of reward mechanisms (Lock et al., 2012; Trail et al., 2012). In addition to traditional methods (e.g., surveys), examining the neural response of fans exposed to different stimuli related to the team can be a valid contribution to better understanding the relationship between the team characteristic and the fan’s feelings.

The ability to remember something is linked to episodic memory and allows human beings to recall specific events about what happened, when and where (Clayton et al., 2007). Episodic memory, in this context, is not just an event, it is the fan’s own event, something personal and personalized, unique from an individual perspective (Clayton et al., 2007).

Memory is a personal construction (Squire and Zola, 1996; Squire, 2009) and each sport fan builds their own memories with the behaviors he/she exhibits, based on context. In fact, sport fans often have routines related to their teams (e.g., following news daily on newspapers and social media, attending live games every week) and these tend to shape their identity levels and behaviors. We assume that fan memories are inseparable from the fan identity level, which may be reflected in neurobehavioral patterns. Therefore, fans’ memories help building the fan identity.

According to Graf and Schacter (1985), the two elements of long-term memory are explicit memory (deliberate remembrance of past experiences) and implicit memory (absenteeism of remembrance). Both are important to shape fan’s identity and behavior. For example, remembering past successful team experiences and blocking failures is often common for sport fans. The ability to remember something is linked to episodic memory and allows human beings to recall specific events about what happened, when and where (Clayton et al., 2007). Episodic memory, enabling people to recall specific events in space (i.e., where), time (i.e., when) and mode (i.e., what), provides meaning to past events (Tulving, 2002). It is not just an event, it is one’s event, personal and personalized, unique from an individual perspective (Clayton et al., 2007). For episodic memory, remembering is a specific concept that requires recollection. Tulving (1993) refers that remembering implies having a conscious perception of an event in the past in which the person participated (e.g., event related to one’s favorite team). Sports fans often remember previous team-related experiences (e.g., games associated features such as the trip to the stadium, eaten food or interactions with others), and associated emotions. In turn, implicit memory is constituted by habit learning, emotional conditioning, priming and perceptual learning, among others (Squire, 2009). It is an unconscious memory that is restored via “how-to” behaviors (Damasio, 2010) and expands consistently due to the fact that what is learned by a person is incorporated in new practices (Squire and Wixted, 2011). Memory is not an individual’s aptitude, is a personal construction (Squire and Zola, 1996; Squire, 2009) and fans build their own with the behaviors exhibited. As fans’ routines tend to shape their identity (Biscaia et al., 2018), one could argue that fan identity is linked team-related memories.

### Fan identity and neural reactions to teams

Research has shown that negative stimuli have a greater preponderance in and neural impact on the brain (i.e., strength, growth, and complexity) than positive stimuli due to a livelihood mechanism (Baumeister et al., 2001; Vaish et al., 2008; Tierney and Baumeister, 2021). This effect has also been confirmed in other areas such as attention (Smith et al., 2003) and imaging studies on the self (Northoff et al., 2006). These studies must be taken into account when studying sports fans, because team brands have different strengths (i.e., status, prestige, on-field success) and fans are exposed to different stimuli that are caused by the match unpredictability (i.e., the uncertainty of the final result often generate emotional uncertainty Chang and Inoue, 2021). Fans’ responses in relation to teams have been increasingly investigated (Havard et al., 2013), with a neural perspective being also incorporated (Hungenberg et al., 2020). For example, Cikara et al. (2011) noted that when baseball fans observe the failures of opposing teams, activity in the limbic system regions increases, while Botzung et al. (2010) noted that activation of dorsal frontoparietal regions is linked to memory accuracy among basketball fans. Also, in a study with football consumers, McLean et al. (2009) suggested that the activation of the anterior cingulate cortex (ACC) is involved in the processing of enjoyment, while Duarte et al. (2017) found activation in the ventral tegmental area (VTA) during the viewing of the football team-related videos. These studies show different neural activation patterns to various stimuli (e.g., failure of opposing teams, memory accuracy, pleasantness, and tribal love/in group bias), allowing a better understanding of neurobehavioral profiles in a defined type of consumer, the sport fan. However, neural reactions among highly identified fans are still to be interpreted because preceding endeavors were comparison of only between fans vs. not fans; (Cikara et al., 2011), and not within fan groups with differing team identity.

Importantly, contrasting one’s relative identity as a fan and the strength of the brand have not yet been explored. Very well-known and strong brands (e.g., professional team brands) have a great importance in the leverage of major emotions and reactions (Alvarez and Fournier, 2016; Veloutsou et al., 2020). Consumers learn about their feelings through experience (e.g., going to matches, using paraphernalia; Sainam et al., 2010), and the sport context is inherently emotional representing a platform for memorable experiences with team brands. Understanding the importance of the role of being a fan when measuring reactions to sport brands is paramount, because fan identity has been suggested to be a central piece for explaining cognitive and affective responses to team- and opponent-related stimuli (Lock and Heere, 2017). Fan identity refers to the importance of the role of being a fan for each individual (Biscaia et al., 2018). It is a role-based measure of fandom that often depends on the individual’s social structures (e.g., family and peers) or demographic elements (e.g., age and social context) (Stets and Burke, 2014). The higher the salience of fan identity in one’s life, the greater the possibility of behavioral choices related to the expectations of such identity (Stryker and Burke, 2000). That is, the role of being a fan implies certain behaviors such as attending games, recommending games to others, purchasing merchandise, or following the team through media (Biscaia et al., 2018). Fans tend to create close relations with sport brands (Kunkel et al., 2020), and there are several factors that shape their role identity (e.g., the prestige of the team and its players; Gwinner and Swanson, 2003). For many fans, particularly those with very high levels of identity, the team is an extension of their identity (Lock and Heere, 2017) allowing them to express themselves in a genuine way (Kunkel et al., 2020; Pedragosa et al., 2023). Based on previous sport consumer research and the remaining need to understand the role of fan identity on individuals’ neural responses to team stimuli, we hypothesize that:

H1: Fans who highly identify with their role will exhibit differential activation patterns in reward and limbic processing regions as compared to those who are very highly identified.

### Brand strength and sport fans

The strength of a brand is more than its market share or its revenues. It also encompasses what exists in the minds of consumers (Krishnan, 1996). That is, measures of sales and market share often represent marketplace manifestations of consumer brand perceptions (Datta et al., 2017; Kunkel and Biscaia, 2020). The strength of a brand is dependent on consumer awareness, mental associations and subsequent behaviors toward that brand (Aaker, 1991; Keller, 1993). For example, Dahlén and Lange (2005) compared ads for two familiar brands and noted that the act of recalling an advertisement favors the purchase of strong brands, with the opposite effect occurring in weak brands. In turn, in sport contexts, team brands are often covered by extensive media exposure meaning that team brand awareness is quite solid (due to an incessant media coverage). Brand associations and fan identification have been suggested to be two of the more important aspects to generate value for team brands (Gladden and Funk, 2002; Bauer et al., 2005; Ross et al., 2008).

In their seminal study about cola beverage preferences (Coca-Cola and Pepsi), McClure et al. (2004) subjected participants to a behavioral taste test during an fMRI. The authors found that knowing the brand changes neural activation patterns, including in areas involved in explicit memory. However, such differentiation only occurred with Coca-Cola. Esch et al. (2012) conducted a study with unfamiliar (weak) and familiar (strong) brands and found that, when exposed to “strong” vs. “weak” brands, consumers activate more areas linked to the executive function, while for “weak” vs. “strong” brands the activation of areas in the brain was found connected with intense and stimulating emotional experiences (also linked to pain and disgust). These two studies suggest that there are neural differences considering the strength of the brand as far as memory is concerned. Nevertheless, there is a lack of knowledge of consumer reactions in the context of sport brands, often known by evoking passionate reactions (Biscaia et al., 2016; Veloutsou et al., 2020) and different fan identity levels.

For “strong” brands the memory recall process is the most solid. In contrast, for “weak” brands, while recovery processes are employed, *ad hoc* processes are the most used. Esch et al. (2012) refer that explicit (i.e., declarative) information differentiates strong from weak brands in terms of familiarity and cognitive associations. They further note that when consumers process brand information, they experience different emotional states, and these should be seen as the main motivators of the brand value (Brakus et al., 2009). The current study also follows up the work of Krishnan (1996) and focus on brands (i.e., teams) with high value and that trigger a number of positive associations among individuals (i.e., fans). As previous studies suggest that strong brands lead to a distinct activation pattern in the brain of consumers (McClure et al., 2004; Esch et al., 2012) and the level of identification is a pivotal aspect to understand the relationship between fans and their teams (Lock et al., 2012), one may assume that may be an overlap of the brain areas activated when very high identified fans of strong brands are exposed to team related videos, videos, a differential pattern may emerge. Thus, based on prior work of Duarte et al. (2017) the following hypotheses are proposed:

H2: Fans of a strong brand will more strongly recruit limbic and memory regions.

H3: When considering the content viewed, there is a dependence of neural activity on contrast levels of fan identity.

## Materials and methods

### Participants

Participants (i.e., fans) were drafted from two teams (FCP: Futebol Clube do Porto; AAC: Associação Académica de Coimbra) competing in *Liga Portugal* at the time of data collection, which was accredited the 6th strongest football league worldwide in 2021 (UEFA, 2022). The current work used a new analysis for different research questions for the fMRI raw data reported in Duarte et al. (2017). Upon the time of data collection (2014/15 season), UEFA ranked FCP in the 16th place in the team European ranking (i.e., considered here a strong brand), while AAC stood at 129th place (i.e., considered here a weak brand) (UEFA, 2016). In addition, FCP had an average stadium attendance of 29,870 spectators per game, while AAC stood by 4,666 spectators (LPFP, 2015). It is worth noting that brand strength was based on performance metrics (e.g., Dahlén and Lange, 2005; Dahlén and Rosengren, 2005) but that does not imply that the teams are perceived the same way by supporters. A total of 61 individuals from both teams were recruited through a snowball sampling technique, given that trust is a key aspect in research conducted in medical facilities (Harsh, 2011) and this experimental study was conducted in a medical school. Eight individuals were excluded 3 did not complete the fMRI and 5 did not respond to at least one of the three conditions: positive, negative, or neutral [see more details in Duarte et al. (2017)]. A final sample of 53 individuals wasincluded in the study, all male, with ages ranging from 20 to 60 years old (*M* = 34.9 ± 10.7 years). Of those, 33 participants were subscribers (i.e., members) of their club (i.e., paying a monthly or annual fee to have discounts on club services) and the length of memberships was above 2 years. All participants attended at least one live game of their teams during the season prior to data collection, and 50 of them attended two or more live games. Also, 42 accompanied their team in, at least, one away game. Study tasks were part of a larger project related to sport and neuroscience.

### Measures and tasks

A short questionnaire including demographic questions and the seven items of the sport spectator identification scale (SSIS) that were validated for the Portuguese context (Theodorakis et al., 2010) was completed by the participants before the fMRI scans. This scale has often been used in past studies due to its brevity and practical utility (Lock and Heere, 2017), and the items are representative of how individuals perceive themselves as fans of their favorite team (Trail and James, 2016; Lock and Heere, 2017; Biscaia et al., 2018). Items capture both an internal (e.g., “How strongly do you see yourself as a fan of [named team]?”) and behavioral perspective (e.g., “How often do you display [named team’s] name or insignia at your place of work, where you live, or on your clothing?”), and are measured on a five-point scale (1 = low identification; 5 = very high identification). In a previous study we showed that the identification scale [SSIS, M(SD) = 4.19(0.74; *n* = 56) and the fanaticism scale (FSFS, M(SD) = 3.16(0.96); *n* = 55] were highly correlated with a shared variance of 64% [*r* (53) = 0.80, *p* < 0.00001] (Duarte et al., 2017).

### Procedures of data collection

During the fMRI scans, participants watched short video streams of acts that caused a goal, and they had to categorize them as positive, negative or neutral. The videos were distinct for fans of FCP and AAC, and aleatory in each session for all subjects. Only the on-field play leading to a scored goal were shown, without any images of spectator reactions, coaches’ behaviors, or any game celebration. The design implied videos of (1) favorite team’s winning or losing moments; (2) winning or losing moments of their favorite team against high rival teams; (3) rival team’s winning or losing moments; and (4) Italian B-series teams (neutral videos). The option for the last type of videos was due to the high likelihood of being unknown goal situations to Portuguese participants, as confirmed by the debriefing after the scanning session. For a full description of the procedures see (Duarte et al., 2017).

### MRI acquisition parameters and data analysis

A 3T Magnetom Trio Tim whole body scanner (Siemens, Erlangen, Germany), using a 12-channel head coil, was used to perform the experiment. A T1-weighted MPRAGE was measured for anatomical identification. The acquisition parameters included a repetition time (TR) of 2530 ms, echo time (TE) of 3.42 ms, resolution 1 mm^3^, flip angle of 7°, matrix size 256 × 256, field of view of 256 × 256 and a slice thickness of 1 mm. Given that EPI-BOLD sequences may suffer distortions from susceptibility artifacts, gradient field maps (GRE) were acquired before each Echo Planar Imaging (EPI) sequence (GRE maps acquired with the same orientation and same field of view, for 54 s and prior to each EPI sequence). Functional information was obtained through EPI sequences acquired parallel to the AC-PC line, covering nearly the whole brain. The acquisition parameters included a slice thickness of 3 mm and voxel size 4 mm^2^, 36 slices, TR 3000 ms, TE 30 ms, flip angle of 90°, matrix size 256 × 256 and FOV of 256 × 256, 190 volumes. Two sequences were acquired for the study. The visual stimulation videos were shown inside the MR scanner by means of an LCD screen (NordicNeuroLab, Bergen, Norway) and the participants viewed the stimuli through a mirror mounted above the participant’s eyes. The monitor had a frequency rate of 60 Hz, a 698.40 mm × 392.85 mm dimension, and was placed ∼156 cm away from the participants’ head. Video audio was provided through headphones, and subjects selected responses using an MR-compatible joystick (Hybridmojo, San Mateo CA, USA).

EPI-BOLD images were undistorted using the GRE maps in the AnatAbacus v1.1 plugin (Breman et al., 2009) for BrainVoyager QX. The pre-processing and analyses were performed in BrainVoyager QX 2.8.2 (Brain Innovation, Maastricht, Netherlands) by using slice scanning time correction; motion correction (the second run was corrected in relation to the first volume of the first run); and filtered in the time domain (two cycles). Anatomical and functional data were co-registered automatically and manually verified, and then transformed to the Talairach space. A General Linear Model (GLM) random effects (RFX) analysis was conducted at group level. The predictors’ model was obtained by convolution of the time course belonging to each condition with a two-gamma hemodynamic response function. Finally, resulting t-maps were corrected for multiple comparisons using false discovery rate (FDR) with a fixed *q*-value lower than 0.01. Reported clusters included at least 25 contiguous voxels.

## Results

### Fan identity and neural reactions

With the exception of one participant who did not answer all items, the levels (SSIS) for participants of both teams varied between 2.14 and 5.00 (*M* = 4.09; SD = 0.76). We divided the pool of participants in two groups (equal in size) of “high” (SSIS ≤ 4.14, *M* = 3.49, SD = 0.61, *n* = 26) and “very high” (SSIS ≥ 4.29, *M* = 4.68, SD = 0.26, *n* = 26) identity scores. Therefore, they were called fans with high identity (FHI) and fans with very high identity (FVHI), respectively. Taking into consideration the SSIS, there were no significant differences in the fan identity levels [*t*-test, t(50) < 2.01, *p* = 0.70] between the sample of fans of the weak brand (FWB SSIS: *M* = 4.04, SD = 0.76, *n* = 26) and the fans of the strong brand (FSB SSIS: *M* = 4.13, SD = 0.77, *n* = 26).

**FIGURE 1:**
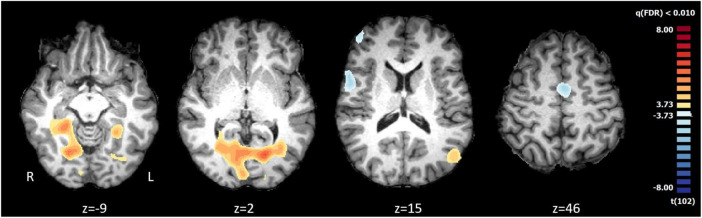
Comparison of brain activity between lower (*n* = 26) vs. higher (*n* = 26) fans accordantly to the identification scores. The statistical map reveals areas of significant differences during the video visualization [*t*(102) > 3.73, *p*(FDR) < 0.01 corrected]. The functional results obtained of comparison of groups were projected in the brain of a single subject in Talairach space.

The t-map [(*t*102) > 3.73, *p*(FDR) < 0.01] of FHI vs. FVHI group comparison during the visualization of the videos (all conditions) showed activations in the visual cortex, lingual gyrus, posterior cingulate, and parahippocampus (the latter being the same regions that were revealed by the contrast weak vs. strong brands) (see Figure 1). On the other hand, the contrast of the groups of very high (FVHI) vs. high identity (FHI) scores [*t*(102) > 3.73, *p*(FDR) < 0.01] showed activation in the inferior frontal gyrus, lateral prefrontal cortex and medial frontal gyrus (regions involved in executive and emotion regulation processes).

There were significant differences during the video’s visualization with activation in neural areas, namely in the lingual gyrus, posterior cingulate, and parahippocampus. The FHI recruited more areas related to visual processing, activating more posterior visual areas. In addition, the FVHI focuses on more areas related to visual and spatial processing related to game viewing, indicating less commitment. Therefore, H1 was supported.

Concerning the contrast level defining fan identity, we predicted a lower response of the cognitive and emotion control network for higher (i.e., the lower level in this study) fan identity levels. These results suggest that brain networks involved in cognitive evaluations and emotional control are differentially activated as a function of the contrast level of fan identity with the team.

### Brand strength and neural reactions

**FIGURE 2:**
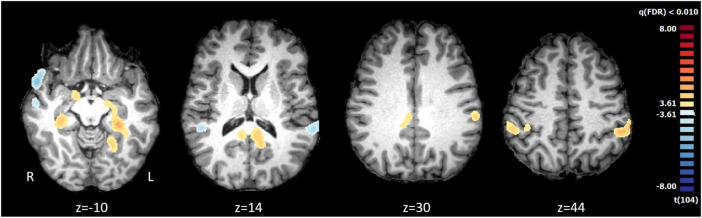
Comparison of weak (AAC, *n* = 26) vs. strong (FCP, *n* = 27) team brands. The statistical map reveals areas of significant differences during the videos visualization [t(104) > 3.61, *p*(FDR) < 0.01 corrected]. The functional results obtained of comparison of groups were projected in the brain of a single subject in Talairach space.

Participants were considered as fans of a weak brand (FWB) or fans of a strong brand (FSB), according the respective team’s success as described in the methods. As noted above, there were no significant differences in fan identity levels [*t*-test, t(50) < 2.01, *p* = 0.70] between the FWB (SSIS: *M* = 4.04, SD = 0.76, *n* = 26) and FSB (SSIS: *M* = 4.13, SD = 0.77, *n* = 26), which allows to proceed the analyses focused on the strength of the brand. The fMRI results of the comparison between FWB (*n* = 26) and FSB (*n* = 27) [*t*(104) > 3.61, *p*(FDR) < 0.01] indicate a differential pattern of activations involving the inferior parietal lobule, entorhinal cortex, posterior cingulate, lingual gyrus, parahippocampus, hippocampus, right amygdala and brainstem (Figure 2). Consequently, H2 was not supported.

We then performed a ROI-based analysis, using the same valence factors. Table 1 shows the mean beta values for both groups (FSB/FWB) during all conditions (positive/neutral/negative videos) in each region, showing that FWB have a distinct neurobehavioral pattern which is common across different videos valences. The stronger recruitment of regions in particular the posterior cingulate involved in visuospatial processing, navigation and memory suggests that FWB are more focused in spatial cognitive analysis of the video scene. Therefore, H3 was not supported because brain activity was indeed distinct across these football fans.

**TABLE 1:** Mean beta values for all factor-level combinations (mean condition effects averaged across FCP and AAC subjects) in the ROI-based analysis.

	Negative videos		Neutral videos		Positive videos	
	**FCP**	**AAC**	**FCP**	**AAC**	**FCP**	**AAC**
Retrosplenium, hippocampus and lingual	0.01	0.21	–0.07	0.23	0.10	0.26
Posterior cingulate	–0.08	0.05	–0.14	0.06	–0.01	0.07
inferior parietal lobule right	–0.07	0.07	–0.19	0.12	–0.03	0,17
inferior parietal lobule left	0.12	0.24	–0.10	0.30	0.06	0.25
Lingual	0.30	0.52	0.23	0.64	0.37	0.63
Entorhinal cortex	–0.23	0.04	-0.45	0.06	-0.13	0.11

A sign test showed that AAC fans elicited statistically significant higher beta values compared to FCP fans (*p* = 0.000008, 18 out of 18 observations revealed positive differences for AAC-FCP). FCP, Futebol Clube do Porto; AAC, Associação Académica de Coimbra.

**FIGURE 3:**
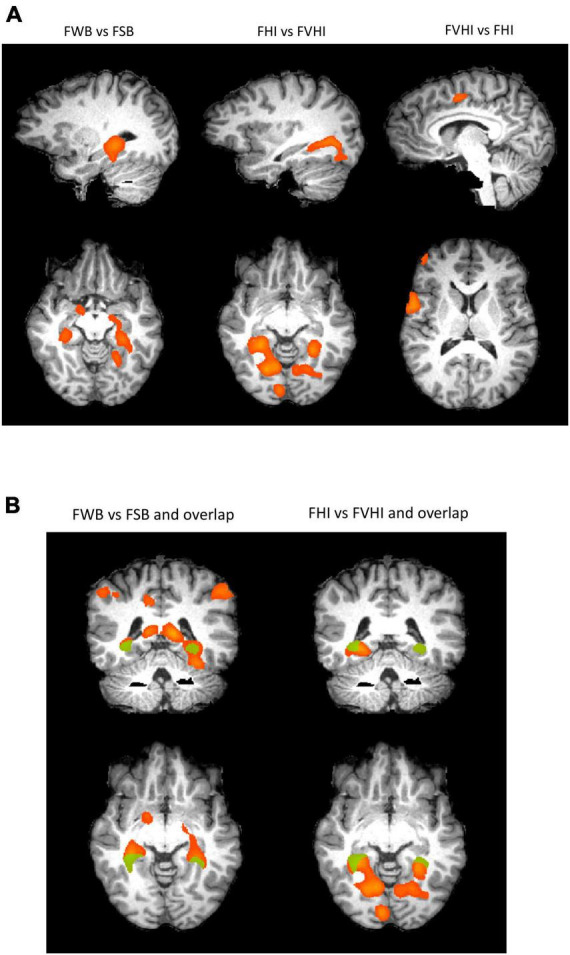
**(A)** Comparison of weak (AAC, *n* = 26) vs. Strong (FCP, *n* = 27) team brands. The statistical map reveals areas of significant differences during the videos visualization [t(104) >3.61, *p*(FDR) < 0.01 corrected]. The functional results obtained of comparison of groups were projected in the brain of a single subject in Talairach space. **(B)** Contrast of FWB vs. FSB and FHI vs. FVHI are shown in relation to their overlap.

The two groups engaged differentially the inferior parietal lobule, entorhinal cortex, posterior cingulate (but to a different extent), lingual gyrus, parahippocampus, hippocampus, and right amygdala. Note that there were no differences in the SSIS scores between fans of both clubs, in spite of the observed neural differences. The posterior cingulate activated for both contrasts of weaker fan vs. strong fan identification and weaker versus strong brand. The results suggest that video content is in general processed differently between both FHI and FVHI and between FWB and FSB, in such a way that activation of posterior cingulate regions is observed in both contrasts. In the FVHI, the regions that process the sensory information introduced by the videos were less active when compared to the FHI. Regarding brand strength, in FSB, fewer but very relevant areas activated than FWB (see Figures 3A, B and statistical maps).

## Discussion

The current study investigates the neural correlates of football fans according to their within-group contrast of identity as fans (high vs. very high), and the relative strength (weak vs. strong) of the team’s brand, which adds to and follows up the prior work of Duarte et al. (2017).

The first major finding this study has identified is that fans with high levels of identification with their team have greater recruitment of temporal and frontal regions related to high level processing of personally relevant information. We found that the fans that scored lower on the identification scale activate more the inferior parietal lobule, entorhinal cortex, posterior cingulate, lingual gyrus, parahippocampus, hippocampus, and right amygdala, when compared with the fans that scored higher. The research has also shown that very high identified fans recruit more anterior regions involved in high level processing and emotion regulation in the temporal and frontal lobe, while fans with less identification activated lower-level limbic regions such as the amydgala. The second key finding is that brain activity is larger in the posterior cingulate for fans of a weak (i.e., AAC) as compared to a strong team (i.e., FCP) brand, suggesting that those who are attached to a weak brand are likely to be engaged in more implicit neural processes. This finding extends past neuroimaging literature examining tribal love (e.g., Duarte et al., 2017) and high vs. low involvement consumers (Dahlén and Lange, 2005; Esch et al., 2012), by providing empirical evidence of different consumer reactions to brands concerning its market strength.

In the current study, we provide evidence for the neural correlates of fan identity (i.e., the importance of the role of being a fan to the individual; Biscaia et al., 2018) with particular attention to aspects of brand strength (weak vs. strong brand). Fan identity refers to the importance of the role of being a fan to the individual (Lock and Heere, 2017; Biscaia et al., 2018) and does not consider the opposing teams or the out-group. Also, brand strength is an important aspect in the sport context given that sport brands are popular worldwide and fans tend to have strong emotional bonds toward them (Bauer et al., 2005). Different patterns of brain activation were shown, mostly temporal and frontal, among individual based on their identity levels. Fans who are very highly identified have greater activation of the neural areas linked to memory and emotions, equivalent to the fan’s feeling “to live the moment” instead of the larger posterior cingulate activation in less involved fans.

In sum the results indicate a brand’s pivotal role in memory and emotion regulation areas. The conscious perception of remembering something means returning to past experiences and relating to the episodic memory (e.g., a trip to the stadium, specific game moments, conversations with other fans, final game result). In turn, implicit memory processes stemming from the posterior cingulate are more prominent in less involved fans.

The current results extend previous literature on fan reactions to sport teams (Duarte et al., 2020; Hungenberg et al., 2020) by providing evidence that brain networks involved in cognitive evaluations and emotional control are differentially activated even among individuals who already exhibit high fan identity level. This suggests that the higher the level of fan identity, the more vulnerable individuals may be to marketing messages because their emotion regulation and control is affected by the high reactivity to positive stimuli. Our findings also corroborate the importance of posterior cingulate as a confirming factor of the link between imagination and action (Schaefer et al., 2006). This finding is important for team brands because the activation of the posterior cingulate is suggested to help the relationship between memory, self-experience, and action (Botzung et al., 2010); and thus, facilitating favorable reactions to the team.

As noted in Figure 1, greater recruitment of areas related to the visual mnemonic processing such as the posterior cingulate were registered for individuals with the lowest but still high levels of fan identity. In contrast, for those with a very high fan identity, we observed a recruitment of more anterior regions, involved and emotion regulation. These results extend previous literature by highlighting the importance of one’s identity in the activation of such neural areas (Esch et al., 2012). Fans who attribute very high importance to the role of being a fan of the team seem to be more “emotional experts,” which likely relates to the resources employed (time, energy, money) and the meaning of the team to their life. While the level of fan identity may limit the ability to generalize these findings to other settings, the results of the current study align with the network of activations related to familiar brands (i.e., higher cognitive functions like imagination of motor tasks, autobiographic memories: Schaefer and Rotte, 2007b). The current findings provide neural evidence that one’s identification with the role of being a fan of a certain team brand plays a role in how he/she behaves, as noted by the recruitment of the regions commonly implicated in memory retrieval (i.e., parahippocampus; Karmarkar et al., 2015a).

The time, energy and money invested by sport fans often shapes their role identity (Biscaia et al., 2018). Over time, an overlap between current integration and a desired integration may occur. That is, individuals likely integrate the brand into their self or expected self (Ahuvia, 1993). It is thought that the lingual gyrus, as an encoder of visual memories (Leshikar et al., 2012), plays an active role in the long-term memory storage process (Zhang et al., 2016). In the current study, the activation of the lingual gyrus could be a reflection of the recall of memories about experiences lived by fans, which highlights the importance of the sport experience design for nurturing the relationship team-fans (Funk, 2017). Thus, the videos in this study seem to have been processed more in an executive logic and semantic memory for fans with very high levels of identification when compared with fans with “only” high levels.

Another key finding is that in the contrast between fans of strong and weak brands (Figure 2) we verified that both groups experience videos differentially at a neural level. The results indicate that individuals who are attached to a weak brand are more likely to be endowed with stronger cognitive appraisal and control mechanisms during video visualization; therefore, less susceptible to marketing messages. The fans of the weak brand (AAC) activate more episodic memory areas, in particular the posterior cingulate, and inferior parietal lobule regions (related to emotional processing and visuospatial and attention integration), which suggest they are more likely to actively cognitive mechanism to regulate their behavior. These results suggest that fans of weak brands might keep a higher level of mnemonic visual recollection control during video appraisal, which might act as a protective mechanism for their frequent unsuccessful events.

On the other hand, fans of the strong brand (FCP) might be more prone to “live the moment” because they are more used to the team’s brand success (e.g., titles, important victories), which makes them proud of the prestige and status it confers. Still, a defeat seems to require a more modest stance to protect themselves (see Table 1). It is also worth noting that for fans of weak brands there seems to be more widespread activation pattern relative to positive videos when compared to neutral and negative videos (see Table 1). This finding contradicts previous studies on psychology highlighting a negative bias (e.g., Tierney and Baumeister, 2021) by suggesting that it is easier for sport fans to forget negative moments and retain only the positive memories that contribute to their role identity as fans of the team. That is, fans always find ways to cope with factors threatening their connection to the teams (Delia, 2019) and one may argue that positive events (e.g., team victories) are more meaningful for sport fans. Consistently, there is the possibility that when individuals process negative information that do not involve a threat, a positive bias rather than a negative bias may occur (Zhao et al., 2017; Verdade et al., 2022).

Findings indicate that video content is processed differently depending on the level of fan identity or the strength of the brand fans are attached to Figures 3A, B. A higher level of fan identity corresponds to a lower neural sensitization to the sensory information perceived in the FVHI/FHI comparison. This fact may be related to the degree of exposure to this type of content. In the comparison between FSB/FWB, concerning brand strength, we observed that fans of the strongest brand have a lower neural activation in implicit processing. That may be related to a deeper, broader, and more global experience, resulting from the brand’s overall exposition power.

## Limitations and future research

There are limitations that should be acknowledged and considered for future studies. First, although the current investigation uses a large sample in fMRI studies, it needs replication. Future studies should recruit and collect data from other groups of highly identified fans of strong and weak brands to test the generalizability of the current findings. Also, additional studies could be designed to gather neural responses across fans with varied levels of identification with their role as fans of their teams (i.e., low, medium, and high). In a similar vein, all participants in the study were males. As reactions to sport teams often vary based on gender (Trail et al., 2008) a balanced sample of male vs. female would be important in additional studies. Second, as noted by Brakus et al. (2009), the strength, intensity, valence, time and space where the brand experience takes place are pivotal for consumers. The findings from the current study were obtained through a study in a laboratory experimental setting. It is possible that this controlled environment may not provide a good overview of the different stimuli elicited while fans attend live or broadcasted events, or interact with their teams in other situations such as social media. Also, additional real-time measures such as cardiac response or eye-tracking (i.e., attention to game situations) may prove to be important to deepen the understanding of fans reactions to team brands in different occasions.

## Conclusion

This study identifies neural correlates of football fans based on the contrast between identity levels (high vs. very high) and the strength of the team brand (weak vs. strong). The results indicate that high fan (relatively lower) identity has a more significant impact on processing sensory information introduced by videos than very high fan identity, which preferentially activate frontal executive and memory regulation regions. As for the brand strength, although groups were similar in identity scores, neural areas of memory and emotion were the ones that most respond to the content depending on the brand’s strength with which the fans are associated. The content displayed was processed differently at a neural level, depending on the fan identity level and the associated brand’s strength. In such an emotional context as the one triggered by sport brands, consumers less identified with a brand were more susceptible to messages as visual processing. In addition, consumers associated with weaker brands activated more areas related to implicit processing.

## Data availability statement

The raw data supporting the conclusions of this article will be made available by the authors, without undue reservation.

## Ethics statement

The studies involving humans were approved by the work “Neural Correlates of Fanhood: The Role of Fan Identity and Team Brand Strength” complies with all the criteria defined by the Ethics Committee. The work has been submitted to the Ethics Committee under reference number CES-UPT-03/01/23. The studies were conducted in accordance with the local legislation and institutional requirements. The participants provided their written informed consent to participate in this study. Written informed consent was obtained from the individual(s) for the publication of any potentially identifiable images or data included in this article.

## Author contributions

All authors listed have made a substantial, direct, and intellectual contribution to the work, and approved it for publication.
